# Integrating Israeli Defense Force (IDF) veterans with disabilities into the workforce: characteristics and predictors

**DOI:** 10.1186/s13584-019-0352-2

**Published:** 2019-12-19

**Authors:** Dan Segev, Miriam Schiff

**Affiliations:** 1 Hebrew University, Paul Baerwald School of social work and social welfare, Mount Scopus, Jerusalem, Israel; 2 Rehabilitation Department, Israel Ministry of Defense, Petach Tikva, Israel

**Keywords:** Rehabilitation, Veterans with disabilities, PTSD, Integration into the workforce, Israel Defense Force (IDF)

## Abstract

**Background:**

According to the ICF Model, the central goal of rehabilitation is the returning of persons with disabilities to an active and fruitful life within society. The Israel Ministry of Defense Rehabilitation Department’s occupational rehabilitation program includes assessment, professional guidance, training, and assistance integrating into employment.

**Aim:**

Examining predictors for the integration of Israel Defense Force veterans with disabilities into the workforce.

**Methods:**

All 1416 male veterans with disabilities who served in the military, were injured during their service, and were treated by the Israel Ministry of Defense Rehabilitation Department between 2001 and 2006 were included in this study. Data collection was based on computerized administrative data in the Ministry of Defense. The data was collected with no identifying details.

Predictive variables were: health condition, personal factors, participation factors, level of functioning and structure, and variables relating to the local environment.

**Results:**

Compared to a physical disability, mental and mixed (physical and mental) disabilities reduce the odds for integration into the workforce. Higher education at the time of the injury is an additional predictor for integration into the workforce. Participation in a rehabilitation track for further education to acquire a profession is one of the main predictors for integration into the workforce.

**Conclusion:**

The results may enable developing more accurate intervention plans, with more efficient use of resources, including consolidation of existing information systems and monitoring the processes and outcomes of rehabilitation.

## Introduction

Since its establishment, the State of Israel has been morally, ethically and legally obligated to care for their veterans with disabilities, who have been injured during the course of their service in the Israel Defense Forces (IDF) [1]. This obligation was expressed in the 1959 “Disability Law (Remuneration and Rehabilitation)” that grants rights to the veterans with disabilities through medical treatment, housing payment, and rehabilitation and which places their integration into the workforce at the center of this process [2] p. 283. The role of rehabilitating the veterans, with the emphasis on their integration into the workforce, falls to the Ministry of Defense – Rehabilitation Department.

Studies in other countries throughout the Western world, were conducted mainly among the general population with disabilities and not among soldiers [3–5]. Studies conducted among US veterans with disabilities due to injuries during service have dealt mainly with aspects such as integration into the workforce among those suffering from psychiatric illness, substance abuse, and/or post-traumatic stress disorder (PTSD) [6, 7].

In the present study we examined variables that may predict the integration of Israeli Defense Forces (IDF) veterans with disabilities into the workforce. Integration into the workforce was defined as a new job placement or the return to veteran’s previous place of work. The predictive variables were derived from the International Classification of Functioning, Disability and Health (ICF), and will be briefly described below.

### Literature review

Occupational rehabilitation is an intervention intended, first and foremost, to integrate people with disabilities into employment [6, 8].

The Israel Ministry of Defense Rehabilitation Department’s occupational rehabilitation program includes assessment, professional guidance, training, and assistance integrating into employment, all of which are designed to help the individual develop lost or neglected skills so as to return to work or find new employment within the job market. The program may include: Physical treatment, psycho-therapy, social services, professional training, preparatory studies, academic studies, mobility support (e.g. accessibility for wheelchairs, support equipment, wheelchair lift), and mentorship (providing a mentor whose role is to assist in developing social skills) [9].

According to the ICF Model, the central goal of rehabilitation is the returning of persons with disabilities to an active and fruitful life within society [10].

Although, originally, it aimed to describe functioning, disability and health [11, 12], it has also been used as a conceptual model for predicting work status [13, 14] and vocational rehabilitation [15]. For example, a study conducted in Israel that was based on the ICF model among 123 longstanding poliomyelitis cases [14] found that almost 60% of the participants were employed and that only one factor of the *body functions and structures*, i.e., dependency in basic activities of daily living, served as a significant barrier for employment. Similarly, our study focuses on integration into the workforce as the outcome, and drew the predictors based on the ICF components and clinical experience. According to the model, there are a number of categories of variables that can predict certain activities such as integration into the workforce: the health condition of the person with the disability, personal factors, participation factors, level of functioning and structure, and environmental factors. In the present study, these categories will be applied within the context of rehabilitating Israel Defense Forces veterans with disabilities.

#### Health condition factors

According to Chan et al. [10], the health condition of the person with the disability is associated, along with additional variables, with the individual’s ability to integrate into the workforce. Health condition, according to the ICF, is a general term for disease (acute or chronic) or injury [12], disorder, or trauma. In the present study, health condition factors include the *severity of the disability* (i.e. the degree of disability as evaluated by a medical committee), as well as the *type of disability* (i.e. physical, mental, PTSD, combination, and head injury). These conditions differ from those that usually are incorporated under the functions and structure factor [16] (e.g., sleep and attention functioning) because they are more stable and present the health status of the person rather than the impairment from the injury. These were also examined apart from the *functions and structure factor* in light of the criticism of the model that it minimizes the importance of the medical aspect of disability [17]. Previous studies found associations between the severity of the disability and functioning level with employment rates: the higher the degree of disability, the lower the employment rate [18, 19]. For example, a study that was conducted in Israel on 3600 veterans with disabilities found that the rate of employment decreases with greater disability severity [20].

##### Type of disability

Studies Show that when it comes to mental disabilities, the rehabilitation outcomes were less favorable than among those with physical disabilities [21]. Studies examining the associations between Post-traumatic Stress Disorder (PTSD) and employment found that veterans diagnosed as suffering from PTSD integrated less into the workforce and/or were more often homeless than veterans with other types of disabilities [7, 22].

#### Personal factors

Personal factors are part of the contextual factors in the ICF which include personal and environmental factors [12, 13]. In this study, we included age, education acquired prior to and during the rehabilitation program, and military rank, all were found to be associated with integration into the workforce [23]. Other predictors examined were: previous employment experience, circumstances of the injury, and type of military service, i.e., compulsory military service, permanent army service, and the reserves [23, 24]. As for *education*- research studies demonstrate that the level of higher education and previous work experience are predictors for integration into the workforce [7, 25].

##### Environmental factors

According to the ICF model, components of the physical, social and attitudinal environment in which people live and conduct their lives may contribute or impede functioning of people with disabilities [12, 16]. These factors are not universal and need to be adapted to the specific context of the population that is being treated or studied [15]. This study includes variables in the physical (e.g., place of residency) or human environment (e.g., family status that implies support from the significant other) that may assist or impede the person with disability’s integration into the workforce. Specifically, we include under the environmental factor the following variables: *Location of residence* (center or peripheral area based on the Central Bureau of Statistics Peripheral Index); *assistance purchasing an apartment* (yes/no); *assistance modifying the residence for the disability* (yes/no); and, *familial status* (at the time of the injury and at the time of the research). Studies in this field have found that these variables are related to rehabilitation outcomes [7, 26, 27].

#### Participation factors

According to the ICF theoretical model, the participation component carries a great deal of weight when predicting rehabilitation outcomes [12]. There are varying definitions for participation. One definition of participation is involvement in a life situation [12]. Another definition includes participation and activities together as learning and applying knowledge, general tasks and demands, communication, mobility, self-care, and interpersonal interactions and relationships [13]. Chan et al. [10], defined this variable as the person with disabilities’ level of participation in his rehabilitation process. This is the definition adopted in the present study, as we believe that choosing to enter a rehabilitation track and to receiving assistance with medical and/or mobility aids can be seen, in and of itself, as evidence of participation in the rehabilitation process and therefore were examined in this study. There are three types of rehabilitation tracks from which the veteran can choose: rehabilitation track of further education (that will eventually lead to getting a professional job), work placement track, and/or economic independence rehabilitation track. The latter is aimed at integrations into the workforce as self-employed or business owner. The t*ype of rehabilitation track*, examined among US veterans suffering from hearing loss who had participated in a vocational rehabilitation (VR) program, was associated with integration into the workforce. The group of veterans who received academic assistance as part of their rehabilitation program better integrated into the workforce than the group which did not receive academic assistance [28].

##### Rehabilitation support assistance

Assistance with job placement, including referrals and setting up job interviews with employers, was consistently found to be associated with integration into the workforce, whether people with disabilities were receiving help with acquiring higher education or not [28–30].

#### Body function and structure factors

This component in the ICF refers to structure and functions of the body, his/her ability to perform activities, and ability to interact with the environment in daily life [16]. In this study we include under this component variables that reflect the capability of the person with disabilities to function and interact with the environment on a daily basis. One variable was *number of impairments.* This variable can tell a lot about their ability to interact with the environment. This may not reflect the severity of the injury, as the severity may be minimal, but the number of impairments that may impede the personal capacity to interact with the environment can be high (for example facial or body scars). A meta-analysis conducted by Saunders et al. [31], revealed that the greater the number of impairments suffered by the person with a disability, the less likely he was to integrate into the workforce. Other structure factors examined were: *assisted by an attendant* (yes/no); *assisted by a mentor* (yes/no) both reflect the severity of the impairment and the level of difficulty in performing daily activities; and, The *number of appeals on the medical committee’s decision*. The number of appeals reflects the person with disability’s perception that his disability is much more severe than perceived by the medical committee that defines the severity of the disability. Such perception may indicate difficulty functioning and interacting with the environment on a daily basis.

## Objectives

**Fig. 1:**
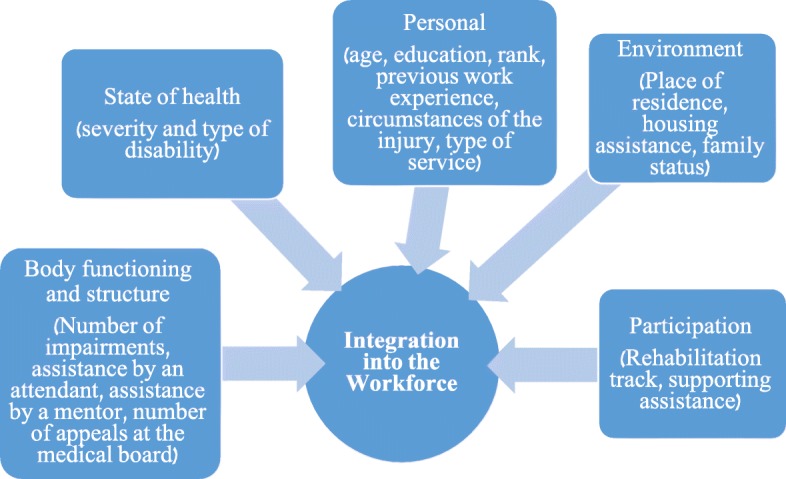
Research model

In this study, we examined predictors for integration into the workforce (the dependent/predicted variable) based on the ICF model. Figure 1 demonstrates the research model and variables examined. Previous studies examined only a portion of the variables and not the entire array. Furthermore, it was never examined within the unique population of Israel Defense Forces veterans with disabilities.

### Research hypothesis

1. Participants with PTSD will be less integrated into the workforce compared to those with other types of disabilities.
2. The higher the level of education at the time of the injury and during the time of this study, the greater his integration into the workforce.
3. The closer the participants’ residence is to the center of the country, the higher the integration into workforce.
4. Health, personal, environmental, participation, and body function and structure variables will be significant predictors for integration into the workforce.

## Methods

### Participants

We examined all 1416 male IDF veterans with disabilities (20% or higher) as a result of various injuries, who were wounded during their service (compulsory military service, permanent army service, and the reserves), and whose disability was recognized by the Ministry of Defense between the years 2001 and 2006. The average age at the time of injury was 24.50 (SD = 6.50). At the time the study was conducted the average age of the participants was 35.70 (SD = 6.60).

### Data collection and research tools

Data collection was based on computerized administrative data in the Ministry of Defense. The data was collected with no identifying details by the researchers and assistant researchers. The study was carried out in strict compliance with the accepted ethical rules and was approved by the Ethics Committee of the authors’ University.

### Data analysis

Data analysis was carried out in two stages. First, the associations between each of the independent variables and the dependent variable, integration into the workforce, were examined using Chi-square and One Way Analysis of Variance (ANOVA) tests. Next, a hierarchical logistic regression analysis was performed to examine the research model.

## Results

Table 1 shows that approximately 62% of the participants are recognized with a physical injury and about 28% with a combined injury (physical and post-trauma). 88% of the participants had an education of up to 12 years of schooling. Of the participants, 77% were employed full-time.

**Table 1:** Type and percent disability, circumstances of the injury, education, and degree of integration into the workforce

	No. of Participants (*N* = 1416)	
	*n*	%
Characteristics of the type of injury		
Physical	734	51.9
Mental	16	1.1
Post-traumatic	55	3.9
Combination (physical and mental)	98	6.9
Combination (physical and post-traumatic)	392	27.7
Head injury	121	8.5
Percent Disability at the time of the study		
Up to 29%	573	40.46
30–39%	247	17.44
40–49%	145	10.24
50–59%	194	13.7
60–69%	93	6.57
70–79%	59	4.17
80–89%	31	2.19
90–100%	27	1.91
100% special ^a^	47	3.32
Circumstances of the injury		
Combat	460	32.5
Training/combat accident	229	16.2
General injury ^b^	374	26.4
Illness	47	3.3
Traffic accident	192	13.6
Terrorism ^c^	114	8.0
Education at the time of the injury		
Up to 12 years	1245	87.9
13–14 years	102	7.2
15+ years	69	4.9
Education at the time of the research		
Up to 12 years	634	44.8
13–14 years	225	15.9
15+ years	557	39.3
Integration into the workforce at the time of the study		
Full-time	1086	76.7
Part-time	103	7.3
Unemployed	227	16

^a^ Special disability includes full paralysis in both legs, full paralysis of half the body, quadriplegic, amputation of two upper or lower limbs, blindness, and certain head injuries

^b^ General injuries – includes injuries such as: Various injuries, injury while playing, damage to objects, etc.…

^c^ Terrorism - Injury as a result of hostile terrorist activity (mainly suicide bombings and explosives)

A chi-Square test was drawn between the type of disability and integration into the workforce. Results show that, in accordance with hypothesis 1, the differences in integration into the workforce between veterans with disabilities recognized with post-trauma and veterans recognized with other type of disabilities are significant, χ^2^(2) = 60.27, *p* < .001, Cramer’s V = .21. The rate of *non*-workers recognized with PTSD is almost double the rate of non-workers recognized for other types of disabilities (24.1 and 12.2%, respectively).

A one-way ANOVA was performed to examine the associations between the age of the participants at the time of the injury and their integration into the workforce. Results showed significant differences between the age of the participants at the time of their injury and their integration into the workforce *F*(2,1413) = 5.50, *p* = .004, *η*^*2*^ = .01. A contrast analysis (Tukey criteria) showed that the average age at the time of injury of those who are currently integrated into full-time work is significantly lower (*Mean* = 24.22, *SD* = 6.27), than the age of those working part-time (*Mean* = 26.21, *SD* = 7.44), and or not working at all (*Mean* = 25.07, *SD* = 6.87) *p* < .001.

### Education level at the time of injury and at the time of the study and integration into the workforce

Results of a one-way ANOVA support hypothesis 2. We found, statistically significant differences between integration into the workforce and the education level at the time of the injury *F*(2,1413) = 8.23, *p* < .001, *η*^*2*^ = .01. Contrast analysis (Tukey criteria) showed that the average years of education at the time of the injury among those integrated into full-time employment is significantly higher (*Mean* = 12.28, *SD* = 1.10) than the average level of education of those working part-time (*Mean* = 12.17, *SD* = 1.12), or not working at all (*Mean* = 11.97, *SD* = .94). The difference in the level of education between full-time and non-working participants was statistically significant at *p* < .001. A similar trend was found when examining the relationship between education at the time of the study and integration into the workforce *F*(2,1413) = 131.85, *p* < .001, *η*^*2*^ = .16. A contrast analysis (Tukey criteria) showed statistically significant differences between integration into the workforce and the education level at the time of the study. The level of education of those working full-time was higher (*Mean* = 13.95, *SD* = 1.8) than the average education of those working part-time (*Mean* = 12.72, *SD* = 1.5), and those not working at all (*Mean* = 12.10, *SD* = .11). The differences between the groups were significant at a level of *p < .01*.

### Associations between environmental factors and integration into the workforce

To examine the relationship between a central place of residence (range from 1 to 5 with a lower number indicating greater distance from center of the country) and integration into the workforce, a one-way ANOVA was performed. We found support for hypothesis 3; participation in the workforce does indeed vary in relation to the distance from or proximity to the center, *F*(2,1413) = 8.52, *p* < .001, *η*^*2*^ = .01.

A contrast analysis (Tukey, criteria) found that participants with full-time employment reside in more centrally located places (*M* = 3.56, *SD* = 1.28) than participants working part-time (*M* = 3.34, *SD* = 1.33), or not working at all (*M* = 3.19, *SD* = 1.24). The differences in proximity to the center between full-time and non-employed participants were significant by *p* < .001.

### Predicting integration into the workforce according to health, personal, environmental, participation, and body function and structure variables

To examine hypothesis 4, a hierarchical logistic regression analysis was conducted. The sets of variables were included according to the order of the model (Enter method). Table 2 presents the results of the analysis. In the first step, state of health variables were entered. The findings indicate that mental disability, head injury, and disability due to post-trauma reduce the odds of integrating into the workforce, compared to physical disability only (*b* = − 1.51, *p* < .001; *b* = − 1.08, *p* < .001; *b* = −.03, *p* < .001, respectively). In addition, an increase in percentage of disability is associated with a decrease in the odds of integrating into the workforce (*b* = −.04, *p* < .001). The health variables in the regression model explain 33% of the variance (Nagelkerke pseudo R^2^). When personal variables were entered in the second step, results show a significant Chi-square change (Chi-square change = 120.09, *df* = 16, *p* < .001). The explained variance also increases from 33 to 44%. A rise in education level increases the odds of integration into the workforce (*b* = .37, *p* < .01). As the age at the time of injury goes up, the odds for integration into the workforce go down (*b* = −.06, *p* < .01). Regarding military rank at the time of the injury, while no difference was found when comparing enlisted men to non-commissioned officers, there was a significant difference in integration into the workforce when comparing officers to enlisted men (*b* = 2.49, *p* < .001). Regarding the circumstances of the injury, the odds for integration into the workforce is lower when the circumstances of the injury are from: training accident (*b* = −.70, *p* < .05), general injury (*b* = − 1.07, *p* < .001), illness (*b* = − 1.77, *p* < .001), and car accident (*b* = −.93, *p* < .01), when compared with a combat injury. No significant difference was found in the integration into the workforce between injuries due to hostile terrorist activity and combat injury.

**Table 2:** Results of the hierarchical logistic regression analysis on the chances for integrating into the workforce (full-time, part-time, and unemployed)

	B	S.E. B	Wald	Exp (B)	95% CI Exp (B)
Step 1: Health Variables					
Fixed	4.59^***^	.27	294.78		
Mental vs. physical disability	−1.51^***^	.38	16.06	.22	[.11, .46]
Combination vs. physical disability	−.43	.26	2.81	.65	[.39, 1.08]
Head injury vs. physical disability	−1.08^***^	.27	16.16	.34	[.20, .58]
Severity of disability	−.04^***^	.004	134.03	.96	[.95, .97]
Post-traumatic disability	−.03^***^	.01	26.26	.98	[.96, .98]
Chi-squared	305.24^***^				
−2 Log likelihood	941.37				
Nagelkerke *R*^2^	.33^***^				
Step 2: Personal Variables					
Education	.37**	.12	9.14	1.44	[1.14, 1.83]
Age at injury	-.06**	.02	7.41	.94	[.90, .98]
Rank			24.67^***^		
NCO vs. enlisted	.43	.39	1.19	1.53	[.71, 3.28]
Officer vs. enlisted	2.49^***^	.61	23.43	18.93	[5.75, 62.25]
Previous work experience	.99^***^	.25	16.07	2.69	[1.65, 4.36]
Circumstances of injury			22.36^***^		
Accident vs. combat	-.70*	.31	5.09	.50	[.19, .62]
General injury vs. combat	-1.07^***^	.30	12.52	.34	[.19, .62]
Illness vs. combat	-1.77^***^	.48	13.83	.17	[.07, .43.]
Driving accident vs. combat	-.93**	.29	10.21	.39	[.22, .70]
Terrorism vs. combat	-.36	.32	1.26	.70	[.38, 1.30]
Type of military service			15.30^***^		
Permanent army vs. compulsory	-1.46***	.40	13.25	.23.	[.11, .51]
Reserves vs. compulsory	-.15	.36	.18	.86	[.42, 1.741]
Δ Chi-Square	120.09^***^				
Chi-Square	425.33^***^				
−2 Log likelihood	821.28				
Nagelkerke R^2^	.44^***^				
Step 3: Environmental Variables					
Housing assistance	-.08	.21	.15	.92	[.62, 1.38]
Peripheral Index	.20**	.07	7.43	1.22	[1.06, 1.40]
Family status			8.91^*^		
Married vs. single	-.86	.50	2.96	.42	[.16, 1.13]
Married with kids vs. single	.57	.31	3.47	1.77	[.97, 3.24]
Divorced vs. single	.25	.34	1.11	1.28	[.81, 2.03]
Δ Chi-Square	15.48^**^				
Chi-Square	440.80^***^				
−2 Log likelihood	805.80				
Nagelkerke R^2^	.46^***^				
Step 4: Participation Variables					
Educational rehabilitation track	2.90^***^	.28	106.13	18.12	[10.44, 31.44]
Economic independence rehabilitation track	.18	.36	.26	1.20	[.60, 2.42]
Work placement rehabilitation track	2.41^***^	.42	33.05	11.08	[4.88, 25.15]
Psycho-therapy assistance	-.27	.23	1.37	.76	[.48, 1.20]
Δ Chi-Square	158.69^***^				
Chi-Square	599.49^***^				
−2 Log likelihood	647.11				
Nagelkerke R^2^	.59^***^				
Step 5: Function and Body Variables					
No. of appeals at medical board	-.19*	.10	3.77	.82	[.68, 1.00]
Number of impairments	.20^***^	.05	15.29	1.22	[1.11, 1.35]
Assistance by attendant	-.84	.44	3.58	.43	[.18, 1.03]
Δ Chi-Square	25.23^***^				
Chi-Square	624.72^***^				
−2 Log likelihood	621.89				
Nagelkerke R^2^	.61^***^				

*CI* Confidence interva

^***^p < .001; ^**^p < .01; ^*^p < .05

In the third step, environmental variables were entered. Improvement in the goodness of fit was found to be significant (Chi-square change =15.48, *df* = 5, *p* < .01). The odds of integrating into the workforce increase as the location of residence gets closer to the center of the country (*b* = .20, *p* < .01). The other environmental variables, assistance in purchasing an apartment and marital status, were not significantly related to integration into the workforce. The rate of explained variance in this step increased from 44 to 46%.

At the fourth step, participation variables were entered (Chi-square change = 158.69, *df* = 4, *p* < .001). The odds of integrating into the workforce among participants who chose a rehabilitation track of further education were high (*b* = 2.90, *p* < .001). The same is true with work placement rehabilitation track (*b* = 2.41, *p* < .001). The rate of explained variance in this step increased from 46 to 59%.

In the fifth step, body function and structure variables were entered (Chi-square change =25.23, *df* = 3, *p* < .001). The odds for integration into the workforce go down as the number of appeals against the decisions of the medical committee go up (*b* = −.19, *p* < .05). On the other hand, the greater the number of impairments, the greater the odds of integration into the workforce (*b* = .20, *p* < .001). The rate of explained variance in this step increased from 59 to 61%.

## Discussion

Coping with a disability can present many difficulties with relationships and one’s self-image, and is occasionally associated with economic hardship [5, 32]. The findings of this study confirm the ICF model [33]. Findings show that rehabilitation outcomes are dependent not only on the type and severity of the injury, but also on a range of variables related to the individual and the environment, as reflected in the research model. Health variables were found to play a significant role in predicting integration into the workforce. When compared with physical disabilities, all other types of disabilities (mental, integrated, post-trauma, head injury) lower the likelihood of integration into the workforce. Furthermore, an increase in the percentage of disability reduces the chances of integrating into the workforce. These findings are consistent with the findings of previous studies conducted in various parts of the world [3, 19, 21, 22, 34].

A possible explanation for the difficulty of the PTSD group in integrating into the workforce is that PTSD includes functional impairments as part of its definition [35]. Work performance is one of the key functional elements and it includes the need to adapt to a hierarchical setting with rules, deadlines, working in a team, and sticking to goals and outcomes. All of these can be challenging for people with PTSD. For example, a study of US military veterans who fought in Iraq examined the relationship between the intensity of post-traumatic symptoms and a wide range of daily life activities, such as employment, social life, and social adjustment. The findings of that study indicated significant associations between the intensity of post-traumatic symptoms and the impairment of the measured functions [36, 37]. Nonetheless, the findings of the present study show that 76% of veterans with PTSD worked part-time or full-time. Thus, a large part of this group of veterans succeeded in integrating into the workforce. This rate is higher than that found among veterans in other parts of the world [7]. A possible interpretation for this interesting finding may be related to the individual rehabilitation tracks tailored to each person according to his situation and abilities. Furthermore, IDF veterans receive support from the government and strong moral support from Israeli society [38]. Social support was found to be related to improved coping with the disability and better functioning [39–41]. The extensive and varied sources of support that exist in Israeli society may explain the difference in findings between this study and the findings of studies conducted on veterans with PTSD in the United States.

The personal variables show that a younger age at the time of injury was associated with greater odds for integration into the workforce than an older age although the effect was small. While these associations are common (e.g., [42]), their explanations are not straightforward. One explanation is the better elasticity of the body and better coping with the challenges in the rehabilitation processes at a younger age. Another explanation is that when the injury occurs at a younger age, the person has not yet acquired a profession, and thus can adapt to various types of jobs while at an older age, the integration into the workforce sometimes requires a change in career. These and other potential explanations should be examined in future studies.

Education at the time of injury is positively associated with integration into the workforce. Similar findings were seen with education levels at the time of the study: The integration of participants with higher education at the time of the study into the workforce was better than among those with a lower level of education at the time of the study. These findings are supported in the research literature [7, 43]. For example, the studies of Crisp and Pester-DeWan [23, 24] found that one of the variables associated with better occupational rehabilitation outcomes was the level of education acquired before entering the rehabilitation program.

As for circumstances of the injury- injury during combat was associated with a greater likelihood of integrating into the workforce compared to injuries under other circumstances. One explanation is that this type of injury may be associated with greater self-esteem, as it is considered a national injury and a national challenge rather than an individual and familial misfortune, and it can raise public support more than other types of injuries [44]. Higher levels of self-esteem are associated with better vocational rehabilitation outcomes [45].

The findings of this study show a positive association between military rank and integration into the workforce. Officers, in contrast to enlisted men and non-commissioned officers, have higher odds of integration into the workforce. These findings may be understood as follows: The higher the rank, the more was required from him as a soldier, the more jobs he was assigned, and the more responsibility he had, including for subordinates. The roles played by officers, their advanced training, and the experience they have accumulated may help them cope with the changes and adaptations required as a result of their injury. In addition, officers usually acquire various training and skills that may assist them in integrating into a wide range of fields [46].

Environmental variables, particularly a central place of residence, are associated with better integration into the workforce. This is similar to the findings in the research literature [47, 48]. A central place of residence has advantages that include, among other things, better accessibility and shorter distances to employment centers [49]. Thus, it can be argued that the greater difficulty among IDF veterans with disabilities in the periphery to integrate into the workforce is more a reflection of the general societal situation than any characterization of this population. At the same time, this difficulty is likely to worsen in peripheral areas due to their disabilities and the need to properly adapt workplaces to their limitations.

The participation variables carry considerable weight when predicting rehabilitation outcomes [10]. Scherer & Glueckauf [50] understand the variables of participation as the level of involvement of the individual in his own life, such as education, employment, and parenthood. In the present study, one of the main predictors of integration into the workforce is the veteran’s choice of rehabilitation track. Specifically, findings of this study, in accord with findings in other studies, show that a rehabilitation track of further education or work placement rehabilitation track, are very important element for integrating into the workforce [24, 51, 52]. It seems that the choice given to every veteran with a disability to select the rehabilitation track that best fits his needs and wants, raises his self-efficacy which is a highly significant predictor for successful rehabilitation [53] #121. Such an interpretation should be further explored in future studies.

## Limitations

Although the study is based on a large sample which includes all IDF veterans with disabilities who were recognized for their disability over a relatively long period of time, it is limited to the period 2001 to 2006. The rationale for choosing these years was the understanding that the rehabilitation processes takes about 5 years, and sometimes even longer, and that all participants in the study should be several years post-completion of their rehabilitation and thereby potentially able to integrate into the workforce. This rationale, however, does not allow us to examine outcomes of changes within the Rehabilitation Department that have occurred in recent years.

In addition, the study is based on a secondary data analysis. Therefore, the measurements of some of the variables could provide only partial information.

## Conclusion

The study’s findings add to the empirical knowledge as well as validate the ICF model as a basis for understanding rehabilitation outcomes. They emphasize the importance of personal and participation factors in this model. They also identify specific risk factors for successful integration into the workforce: older age while injured, lower level of education, circumstances of injury other than combat or terrorism, diagnoses of PTSD, peripheral residential areas, and the choice of rehabilitation track other than education or Work placement rehabilitation track. Each factor by itself indicates a potential challenge within the rehabilitation process. Having several of those risk factors should serve as an alarm for the social workers in the department of rehabilitation to pay special attention and design specific interventions for each profile. The results of the study show how systematic collection of administrative data may enable planning intervention and treatment more accurately, and conducting ongoing monitoring of the processes and outcomes of rehabilitation of veterans with disabilities, especially among those with greater risk for less successful rehabilitation outcomes.

## Data Availability

Data is confidential and therefore cannot be available.

## References

[1] Araten-Bergman T, Tal-Katz P, Stein M (2015). Psychosocial adjustment of Israeli veterans with disabilities: does employment status matter?. Work.

[2] Knesset JI. Disabled law (Remuneration and rehabilitation). In: Book I, editor. Jerusalem: Knesset; 1959. p. 259. [combined version].

[3] Brickham DM. Predicting vocational rehabilitation outcomes for people with alcohol abuse/dependence: an application of Chi-squared automatic interaction detector. In: Psychology R, editor. Ann Arbor: The University of Wisconsin; 2012.

[4] Carter EW, Austin D, Trainor AA (2012). Predictors of postschool employment outcomes for young adults with severe disabilities. J Disabil Policy Stud.

[5] Migliore A, Butterworth J, Zalewska A (2014). Trends in vocational rehabilitation services and outcomes of youth with autism: 2006-2010. Rehabil Couns Bull.

[6] Frain MP, Bishop M, Bethel M (2010). A roadmap for rehabilitation counseling to serve military veterans with disabilities. J Rehabil.

[7] Resnick SG, Rosenheck RA (2008). Posttraumatic stress disorder and employment in veterans participating in veterans health administration compensated work therapy. J Rehabil Res Dev.

[8] Patterson JB, Bruyere SM, Szymanski EM, Jenkins W, RM P, Patterson JB (2012). Philosophical, historical, and legislative aspects of the rehabilitation counseling profession. Rehabilitation counseling: basics and beyond (5th Ed).

[9] VandenBos GR, American Psychological A (2007). APA dictionary of psychology.

[10] Chan F, Gelman JS, Ditchman N, Kim J-H, Chiu C-Y. The World Health Organization ICF model as a conceptual framework of disability. Understanding psychosocial adjustment to chronic illness and disability: a handbook for evidence-based practitioners in rehabilitation. New York: Springer Publishing Co; 2009. p. 23–50.

[11] Escorpizo R, Reneman MF, Ekholm J, Fritz J, Krupa T, Marnetoft S-U (2011). A conceptual definition of vocational rehabilitation based on the ICF: building a shared global model. J Occup Rehabil.

[12] World Health Organization (2001). International classification of functioning, disability and health: ICF.

[13] Wasiak R, Young AE, Roessler RT, McPherson KM, van Poppel MNM, Anema JR (2007). Measuring return to work. J Occup Rehabil.

[14] Zeilig G, Weingarden H, Shemesh Y, Herman A, Heim M, Zeweker M (2012). Functional and environmental factors affecting work status in individuals with longstanding poliomyelitis. J Spinal Cord Med.

[15] Momsen AH, Stapelfeldt CM, Rosbjerg R, Escorpizo R, Labriola M, Bjerrum M (2019). International classification of functioning, disability and health in vocational rehabilitation: a scoping review of the state of the field. J Occup Rehabil.

[16] Prodinger B, Cieza A, Oberhauser C, Bickenbach J, Üstün TB, Chatterji S (2016). Toward the international classification of functioning, disability and health (ICF) rehabilitation set: a minimal generic set of domains for rehabilitation as a health strategy. Arch Phys Med Rehabil.

[17] Rimmerman A (2014). Social inclusion of people with disabilities national and international perspectives.

[18] Gannon B (2005). A dynamic analysis of disability and labour force participation in Ireland 1995-2000. Health Econ.

[19] Oguzoglu U (2009). Severity of work disability and work.

[20] Schnurr Y, Ziv A, Brodeski J, Neon D (2017). Aging among the IDF veterans with disablity and its implications for the need for services.

[21] Chan JY, Keegan JP, Ditchman N, Gonzalez R, Zheng LX, Chan F (2011). Stigmatizing attributions and vocational rehabilitation outcomes of people with disabilities. Rehabil Res Policy Educ.

[22] Quinn A (2008). A person-centered approach to the treatment of combat veterans with posttraumatic stress disorder. J Humanist Psychol.

[23] Pester-DeWan J (2003). Biodata predictors of performance: veterans in the vocational rehabilitation and employment program at the Veterans Administration in San Diego, California.

[24] Crisp R (2005). Key factors related to vocational outcome: trends for six disability groups. J Rehabil.

[25] Razzano LA, Cook JA, Burke-Miller JK, Mueser KT, Pickett-Schenk SA, Grey DD (2005). Clinical factors associated with employment among people with severe mental illness - findings from the employment intervention demonstration program. J Nerv Ment Dis.

[26] Bevan S, Zheltoukhova K, Summers K, Bajorek Z, O’Dea L, Gulliford JJL (2013). Life and employment opportunities of young people with chronic conditions.

[27] Hoge CW, Auchterlonie JL, Milliken CS (2006). Mental health problems, use of mental health services, and attrition from military service after returning from deployment to Iraq or Afghanistan. JAMA.

[28] Boutin DL (2011). Effective vocational rehabilitation services for military veterans. J Appl Rehabil Couns.

[29] Moore CL, Alston RJ, Donnell CM, Hollis B (2003). Correlates of rehabilitation success among African American and Caucasian SSDI recipients with mild mental retardation. J Appl Rehabil Couns.

[30] Rogers JB, Bishop M, Crystal RM (2005). Predicting rehabilitation outcome for supplemental security income and social security disability income recipients: implications for consideration with the ticket to work program. J Rehabil.

[31] Saunders JL, Leahy MJ, McGlynn C, Estrada-Hernandez N (2006). Predictors of employment outcomes for persons with disabilities: an integrative review of potential evidenced-based factors. J Appl Rehabil Couns.

[32] Migliore A, Butterworth J (2008). Trends in outcomes of the vocational rehabilitation program for adults with developmental disabilities: 1995-2005. Rehabil Couns Bull.

[33] W.H.O (2002). Towards a common languge for functioning, disability and health ICF.

[34] Tsai J, Rosenheck RA (2013). Examination of veterans affairs disability compensation as a disincentive for employment in a population-based sample of veterans under age 65. J Occup Rehabil.

[35] Manderscheid RW (2007). Helping veterans return: community, family, and job. Arch Psychiatr Nurs.

[36] Savoca E, Rosenheck RA (2000). The civilian labor market experiences of Vietnamera veterans: the influence of psychiatric disorders. J Ment Health Policy Econ.

[37] Shea MT, Vujanovic AA, Mansfield AK, Sevin E, Liu F (2010). Posttraumatic stress disorder symptoms and functional impairment among OEF and OIF National Guard and Reserve veterans. J Trauma Stress.

[38] Tal-katz P, Araten T, Rimmerman A (2011). Israeli policy toward veterans with disabilities: a snapshot and insights of the proposed reform. J Soc Work Disabil Rehabil.

[39] Berkman LF, Cohen S, Syme SL (1985). The relationship of social networks and social support to morbidity and mortality. Social support and health.

[40] Devine D, Parker PA, Fouladi RT, Cohen L (2003). The association between social support, intrusive thoughts, avoidance, and adjustment following an experimental cancer treatment. Psychooncology.

[41] Snead SL, Davis JR (2002). Attitudes of individuals with acquired brain injury towards disability. Brain Inj.

[42] Ahonle ZJ, Barnes M, Romero S, Sorrells AM, Brooks GI. State-Federal vocational rehabilitation in traumatic brain injury: what predictors are associated with employment outcomes? Rehabil Couns Bull; 2019.10.1177/0034355219864684.

[43] Hess DW, Ripley DL, McKinley WO, Tewksbury M (2000). Predictors for return to work after spinal cord injury: a 3-year multicenter analysis. Arch Phys Med Rehabil.

[44] Barak A, Leichtentritt RD (2015). Ideological meaning making after the loss of a child: the case of Israeli bereaved parents. Death Stud.

[45] Hartke RJ, Trierweiler R (2015). Survey of survivors’ perspective on return to work after stroke. Top Stroke Rehabil.

[46] Avrahami A, Dewik Z, Dangor N, Rimerman A (1997). Rehabilitation specialization in social work: definition, theory, skills and professional coordination (Hebrew).

[47] François DR, Marius T, Yan K, Paul V (2002). Landscaping and house values: an empirical investigation. J Real Estate Res.

[48] Yates J (2002). A spatial analysis of trends in housing markets and changing patterns of household structure and income.

[49] Zibel N (2008). Peripherality index of local authorities 2004 - new development.

[50] Scherer MJ, Glueckauf R (2005). Assessing the benefits of assistive technologies for activities and participation. Rehabil Psychol.

[51] Ben-David D, Ben-David (2010). A macro perspective of Israel’s society and economy; Public expenditures - A look at Israel’s national priorities; Israel’s education system - An international perspective and recommendations for reform. State of the nation report: society, economy and policy in Israel 2009.

[52] Kimhi A, Ben-David D (2012). Labor market trends: employment rate and wage disparities. State of the nation report - society, economy and policy in Israel 2011–2012.

[53] Castillo YA, Fischer JM. Self-employment as career choice for people with disabilities: Personal factors that predict entrepreneurial intentions. J Rehabil. 2019;85(1):35–43

